# *Medicago sativa* and *Medicago truncatula* Show Contrasting Root Metabolic Responses to Drought

**DOI:** 10.3389/fpls.2021.652143

**Published:** 2021-04-21

**Authors:** Andres Echeverria, Estíbaliz Larrainzar, Weiqiang Li, Yasuko Watanabe, Muneo Sato, Cuong Duy Tran, Jose A. Moler, Masami Yokota Hirai, Yuji Sawada, Lam-Son Phan Tran, Esther M. Gonzalez

**Affiliations:** ^1^Institute for Multidisciplinary Research in Applied Biology (IMAB), Public University of Navarra, Pamplona, Spain; ^2^State Key Laboratory of Cotton Biology, Department of Biology, Institute of Plant Stress Biology, Henan University, Kaifeng, China; ^3^Henan Joint International Laboratory for Crop Multi-Omics Research, Henan University, Kaifeng, China; ^4^Stress Adaptation Research Unit, RIKEN Center for Sustainable Resource Science, Yokohama, Japan; ^5^Metabolic System Research Team, RIKEN Center for Sustainable Resource Science, Yokohama, Japan; ^6^Agricultural Genetics Institute, Vietnam Academy of Agricultural Sciences, Hanoi, Vietnam; ^7^Department of Statistics, Computing and Mathematics, Public University of Navarra, Pamplona, Spain; ^8^Institute of Research and Development, Duy Tan University, Da Nang, Vietnam; ^9^Institute of Genomics for Crop Abiotic Stress Tolerance, Department of Plant and Soil Science, Texas Tech University, Lubbock, TX, United States

**Keywords:** plant roots, drought stress, sucrose, sucrose synthase, raffinose, flavonoids, *Medicago*, metabolomics

## Abstract

Drought is an environmental stressor that affects crop yield worldwide. Understanding plant physiological responses to stress conditions is needed to secure food in future climate conditions. In this study, we applied a combination of plant physiology and metabolomic techniques to understand plant responses to progressive water deficit focusing on the root system. We chose two legume plants with contrasting tolerance to drought, the widely cultivated alfalfa *Medicago sativa* (*Ms*) and the model legume *Medicago truncatula* (*Mt*) for comparative analysis. *Ms* taproot (tapR) and *Mt* fibrous root (fibR) biomass increased during drought, while a progressive decline in water content was observed in both species. Metabolomic analysis allowed the identification of key metabolites in the different tissues tested. Under drought, carbohydrates, abscisic acid, and proline predominantly accumulated in leaves and tapRs, whereas flavonoids increased in fibRs in both species. Raffinose-family related metabolites accumulated during drought. Along with an accumulation of root sucrose in plants subjected to drought, both species showed a decrease in sucrose synthase (SUS) activity related to a reduction in the transcript level of *SUS1*, the main *SUS* gene. This study highlights the relevance of root carbon metabolism during drought conditions and provides evidence on the specific accumulation of metabolites throughout the root system.

## Introduction

Grain and forage legumes representapproximately 15% of the worldwide cultivated land, and are one of the most important protein sources for human diet and animal feed (Vance et al., 2000; Daryanto et al., 2015). Their nitrogen fixation capacity, in symbiosis with soil *Rhizobium* bacteria, allows them to be considered environmentally sustainable crops (Graham and Vance, 2003). However, their variable yield under water deficit (WD) conditions limits widespread legume cultivation. To meet global food and feed requirements, considering current climate change scenarios, it is important to comprehensively understand how plants respond and adapt their metabolism when water becomes a limitation (Anjum et al., 2017; Abdelrahman et al., 2018b; Zandalinas et al., 2018; Castañeda et al., 2019). The adaptive response of the plant root system is an essential component of plant responses to WD. Nevertheless, most studies have focused on WD effects on the aerial part, leaving the roots largely unexplored.

Based on their morphology, there are two types of root systems: (i) the tap root type presents the main tap/primary root (tapR) that grows vertically and has lateral branches called fibrous roots, and (ii) the fibrous root type, which has mainly branching fibrous/secondary roots (fibRs) (Tian et al., 2014; Wasaya et al., 2018; Castañeda et al., 2019). Alfalfa *Medicago sativa* (*Ms*) is among the most widely cultivated forage legume species in temperate climates, occupying around 30 million hectares worldwide (Annicchiarico et al., 2011, 2015; Ray et al., 2015; Zhang et al., 2015). Being typically cultivated in arid and semi-arid regions, alfalfa, a perennial specie, can reach deep soils to access water (Humphries and Auricht, 2001; Radovic et al., 2009; de Smet et al., 2012; Quan et al., 2016; Huang et al., 2018), and arrest vegetative growth in case of water deficit (Sheaffer et al., 1988; Zhang et al., 2019). Contrastingly, *Medicago truncatula* (*Mt*) originated in the Mediterranean area and is cultivated as an annual crop in countries such as Australia (Young and Udvardi, 2009). *Mt* is a diploid and model legume species (Barker et al., 1990; Young and Udvardi, 2009), phylogenetically related to alfalfa (Aubert et al., 2006; Phan et al., 2007). As it is naturally present in Mediterranean semi-arid regions, *Mt* is considered a relatively drought-tolerant species compared with *Ms* (Zhang et al., 2014). At the root system level, *Ms* is an example of the tapR type, as it develops a main tapR system to explore deeper soil regions (Humphries and Auricht, 2001; de Smet et al., 2012; Araújo et al., 2015; Huang et al., 2018). In contrast, *Mt* can be included in the fibR category, with a less developed tapR and highly developed fibR system (Schultz et al., 2010; Bourion et al., 2014; Castañeda et al., 2019). Indeed, annual plants typically present low-density root with high nitrogen levels, whereas perennial plants favor persistent dense root systems (Roumet et al., 2006). Concerning shoot growth, *Ms* and *Mt* also differ, as *Ms* shoots grow vertically (Humphries and Auricht, 2001), while *Mt* shoots develop mostly horizontally (Castañeda et al., 2019). Analyzing the different strategies of these phylogenetically related species is needed to further understand the plant metabolic acclimation strategies to water scarcity.

In this study, we hypothesized that drought tolerance differences between *Ms* and *Mt* might be mediated by metabolic changes occurring at the root level. We examined the different root system morphologies of *Ms* and *Mt* and explored the metabolic acclimation of leaves, tapRs and fibRs to WD conditions using non-targeted liquid chromatography with tandem mass spectrometry (LC/MS-MS). Moreover, root carbon metabolism was thoroughly analyzed with particular attention to sucrose synthase (SUS) activity and expression of corresponding genes. Results showed that *Ms* and *Mt* employed different root growth strategies to deal with WD conditions, as suggested by differential metabolic WD responses in both tapRs and fibRs. Although a specific accumulation of raffinose family oligosaccharides was found in the *Mt* root system, a general downregulation of root sucrose catabolism associated with a decrease in SUS activity was observed in the roots of both *Medicago* species.

## Materials and Methods

### Plant Materials and Growth Conditions

Two *Medicago* species, *M. sativa* L. cultivar Sitel (*Ms*) and *M. truncatula* Jemalong A17 (*Mt*), were used in this study. *Ms* and *Mt* seeds were germinated as previously described (Garcia et al., 2006). Seedlings were transferred to one-liter pots containing perlite:vermiculite (2:5, v/v) and grown under controlled environmental conditions (22°C/18°C day/night temperature, 12 h photoperiod, 70% relative humidity, 500 μmol m^–2^ s^–1^). Plants were watered using Evans solution as described by Castañeda et al. (2019).

### WD Stress Treatment

Fifty-seven-day-old *Ms* and *Mt* plants were randomly separated into three sets containing seven plants each per species. The first set, control plants (C), was maintained under well-watered conditions throughout the experiment, while the other two sets were exposed to WD stress by withholding irrigation. After 6 days of water withholding, the leaf water potential (Ψ_w_) was measured daily to monitor the water status of stressed plants. The first fully expanded leaf was used to measure Ψ_w_ in a pressure chamber (3000 Series Plant Water Status Consoles, Soil Moisture Equipment, Santa Barbara, United States; Scholander et al., 1965). *Ms* and *Mt* plants were harvested at their late vegetative stage (approx. 8 weeks post-germination) when their leaf water potential reached approximately −1.5 and −2.5 MPa for moderate (MD) and severe (SD) WD stress, respectively. C samples showed an average Ψ_w_ close to −1 MPa. During the drought period, plants progressively consumed the reserves of water and nutrients in the vermiculite/perlite substrate simulating field-like conditions, and no visual symptoms of nutrient deficiency were observed (Hu and Schmidhalter, 2005). During harvest, tapRs were separated from the fibRs, and these root fractions and leaves were immediately frozen in liquid nitrogen and stored at −80°C until further analysis. Dry weight (DW) was obtained after drying plant material at 70°C for 48 h. Then, water content (WC) from the different tissues was calculated using the following equation: WC = (FW–DW)/(FW × 100), where FW represents fresh weight. Transpiration was measured daily by weighing the pots 15 min and 24 h after irrigation. The difference between both values was the total amount of water lost each day. Plant WD was controlled daily by measuring stomatal conductance (nmol m^–2^ s^–1^) in the oldest fully formed leaves using an AP4 leaf porometer (Delta-T Devices, Cambridge, United Kingdom).

### Determination of Enzyme Activities

Aliquots of frozen tapRs and fibRs (≈ 0.3 and 0.4 g, respectively) were homogenized to a fine powder with liquid nitrogen. Extraction buffer [50 mM MOPS pH 7.5, 10 mM MgCl_2_, 0.1% (v/v) Triton X-100, 10 mM DTT β-mercaptoethanol, 1 mM EDTA, 20 mM KCl, 2.5% PVPP, and 2 mM PMSF supplemented with a protease inhibitor cocktail tablet] was used in the process, and samples were centrifuged for 30 min at 10,625 × *g* at 4°C. Protein content was determined in the crude extract as previously described (Bradford, 1976) using BSA as the protein standard. The crude extract was desalted using BioGel P-6 DG Desalting Gel (Bio-Rad, Hercules, CA, United States) equilibrated with desalting buffer [250 mM MOPS (pH 7.5), 100 mM KCl, and 50 mM MgCl_2_]. The desalted extract was used to determine the activities of UDP-sucrose synthase (SUS, EC 2.4.1.13), alkaline invertase (INV, EC 3.2.1.26), and glucose-6-phosphate dehydrogenase (G6PDH, EC 1.1.1.49). All enzyme activities were assayed as described by Castañeda et al. (2019).

### Metabolomic Analysis

Leaf and root samples of *Ms* and *Mt* were lyophilized using a freeze-drying machine (dry chamber, DRC-1000; freeze-drying instrument, FDU-2100; EYELA Corporation, Tokyo, Japan). The lyophilized samples were weighed using a balance (AP324W, Shimadzu Corporation), and each sample was transferred to a 2 mL tube containing a zirconia bead (5 mm diameter). Extraction solvent containing 0.1% (v/v) formic acid in 80% (v/v) methanol with internal standards (8.4 nM lidocaine, 210 nM 10-camphorsulfonic acid) was added to the tube (1 mg mL^–1^), and the metabolites were extracted in a bead-shaker (Shake Master NEO, Biomedical Science) for 2 min at 1,000 rpm. Using a liquid handling system (Microlab STAR plus, Hamilton Corporation, Mount Airy, MD, United States), the extracted solutions were dried and re-dissolved in LC-MS grade water (FUJIFILM Wako Pure Chemical Corporation, Osaka, Japan). The re-dissolved solution was filtered (AB-0564, Thermo Fisher Scientific), and 1 μL solution containing 100 ng of sample was then subjected to LC coupled with tandem quadrupole MS (LC-QqQ-MS) (NexeraMP-LCMS8050, Shimadzu Corporation, Kyoto, Japan) to measure metabolites.

Using flow injection analysis, selected reaction monitoring conditions of 501 metabolites were optimized, and the retention times (RT) were assigned using LC-QqQ-MS (Supplementary Table 1, selected reaction monitoring and RT; Supplementary Table 1, LC conditions; Supplementary Table 1, QqQ-MS conditions). The raw peak area values of 501 metabolites were collected using LabSolution software (Shimadzu Corporation, Kyoto, Japan) and converted using the Reifycs Analysis Base File Converter^1^. The peak area values of LC-QqQ-MS data were calculated using MRMPROBS (Tsugawa et al., 2013)^2^. The raw data matrix was summarized in Supplementary Table 1, and the intensity data matrix of all metabolites detected was shown in Supplementary Table 1. Normalized intensity data were determined by dividing raw data with intensity data (Supplementary Table 2).

### Phylogenetic and Expression Analyses of the *SUS* Family

Genomic and protein sequences of *Mt*, *Glycine max*, *Oryza sativa*, and *Arabidopsis thaliana* were collected from Phytozome Version 12^3^. Phytozome BLAST-protein searches^4^ were used to identify the putative *SUS* family members using the protein sequence of *Mt SUS1* (*Medtr4g124660*) as a reference. Amino acid sequences were aligned using ClustalW^5^, and phylogenetic tree construction was performed using the maximum likelihood algorithm in MEGA v10.1 (Kumar et al., 2018). The parameters were as follows: model, WAG (Whelan and Goldman, 2001); bootstrap, 1,000 replicates, and gaps/missing data, partial deletion, as previously described (Xu et al., 2019).

The expression of *SUS1* genes (gene IDs *MS.gene030241* and *Medtr4g124660* for *Ms* and *Mt*, respectively) was quantified by qRT-PCR using the F-box/ankyrin repeat SKIP35-like protein-encoding gene as a reference (gene IDs *MS.gene95033* and *Medtr4g134960* for *Ms* and *Mt*, respectively; Sañko-Sawczenko et al., 2019). cDNA synthesis, qRT-PCR, DNase I treatment, and data analysis were conducted as previously described (Le et al., 2012). The specific primer pairs used in qRT-PCR were listed in Supplementary Table 3. The MtSSPdb database^6^ was queried to extract the relative *SUS* gene expression values in the different *Mt* tissues.

### Statistical Analysis

Data were examined within the SPSS 25.0 package (SPSS Inc., Chicago, IL, United States) using a two-factorial analysis of variance (ANOVA, *P* ≤ 0.05), and the Duncan’s test was applied to determine organ-associated and species-associated effects. Student’s *t*-test was employed when a single comparison was required. Data were shown as means ± standard errors of at least four biological replicates (*n* = 4-7 independent biological replicates). The number of biological repeats used in each analysis was specified in the legend of the corresponding figure and table. Significant interactions of WD with species and organs were shown in Supplementary Table 4. Regarding physiological data, three-organ interactions (leaves, tapRs, and fibRs) were analyzed, whereas for carbon metabolism, two-organ interactions (tapRs and fibRs) were assayed. The data matrix of metabolic profiles was analyzed with R 3.6.2 (The R Foundation for Statistical Computing), using the mixOmics package (Rohart et al., 2017) for principal component analysis (PCA) and partial least square discriminant analysis (PLS-DA). Metabolites were classified according to their functional category using the Kyoto Encyclopedia of Genes and Genomes (KEGG)^7^ database (Kanehisa and Goto, 2000; Kanehisa et al., 2015).

## Results

### Differential Physiological Responses to Water Deficit of Tap and Fibrous Roots

After Ms and *Mt* plants were grown for 8 weeks under controlled environmental conditions, progressive WD was applied by withholding water. To estimate stress levels in both plant species, leaf water potential (Ψ_w_) values were monitored. Moderate WD (MD) conditions were considered with an average of −1.51 ± 0.02 MPa and −1.56 ± 0.03 MPa for *Ms* and *Mt*, respectively. For SD conditions, average Ψ_w_ values of −2.53 ± 0.11 MPa and −2.61 ± 0.06 MPa were found for *Ms* and *Mt*, respectively. Well-watered control plants maintained average Ψ_w_ values of −1.15 ± 0.05 MPa and −1.02 ± 0.01 MPa for *Ms* and *Mt*, respectively.

Stomatal conductance showed a significant decrease between C and WD plants after 4 days of treatment in both *Ms* and *Mt* species, with *Ms* showing significantly higher values than *Mt* under WD until day 7 (Supplementary Figure 1A). In agreement with this result, *Ms* showed significantly higher transpiration than *Mt* during the whole treatment process (Supplementary Figure 1B), and this difference was maintained until the end of the experiment. The average transpiration of C plants was 26.76 ± 1.04 and 19.39 ± 1.14 g for *Ms* and *Mt*, respectively.

**FIGURE 1:**
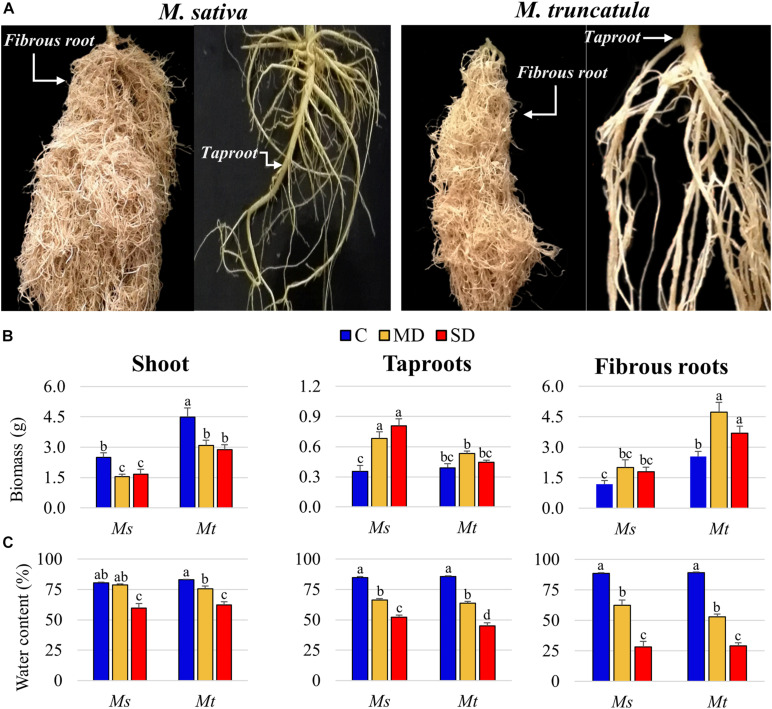
Physiological characterization of the plants. **(A)** Images of 8-week old *Medicago sativa* (*Ms*) (left) and *M. truncatula* (*Mt*) (right) taproot (tapR) and fibrous root (fibR) systems. **(B)** Biomass and **(C)** water content of *Ms* and *Mt* plants under control (C), moderate water deficit (MD), and severe water deficit (SD) conditions. Bars represent means ± SEs (*n* = 7 biological replicates). Different letters indicate significant differences according to a Duncan-test (*P* ≤ 0.05).

To independently analyze the individual responses of the different root system components, *Ms* and *Mt* roots were separated into sections as described in Figure 1A and tapR and fibR samples were collected. Under control conditions, fibR biomass was 3.34 and 6.50 times higher than tapR biomass in *Ms* and *Mt*, respectively (Figure 1B). Regarding plant growth, *Mt* shoot biomass was significantly higher than that of *Ms* at the beginning of the WD treatment. In both species, shoot growth was arrested as soon as stress was applied (Figure 1B). Concerning root biomass, two different trends were observed; in *Ms*, tapR biomass showed a progressive increase as the stress became more intense, whereas in *Mt*, only fibR responded to the treatment (Figure 1B). Regarding WC, shoots showed a significant decline, reaching close to 60% under SD conditions for both species (Figure 1C). Similarly, tapR WC progressively decreased by almost 50% under SD conditions, while the decline observed in fibRs was relatively more severe, with average values of nearly 30% under SD for both plant species (Figure 1C). ANOVA showed a significant interaction between WD treatment and organ type regarding their effect on WC in both *Ms* and *Mt* (Supplementary Table 4), further supporting the differential organ-specific responses observed not only in leaves vs. roots but also in tapRs vs. fibRs.

### Key Metabolic Signatures in Roots of Drought-Stressed Plants Identified by Shotgun Metabolomics

To identify the main compounds involved in root adaptation to drought stress, we analyzed the metabolic profiles of the different *Ms* and *Mt* plant tissues subjected to progressive WD. The soluble metabolite fractions of leaf, tapR, and fibR samples were extracted, and various metabolites were detected and quantified using liquid chromatography coupled with a triple-quadrupole mass spectrometer. This approach led to the quantification of 501 metabolites (Supplementary Table 2), with 287 showing at least a two-fold relative abundance change in SD samples compared with those of C samples (Supplementary Table 5).

Principal component analysis discriminated the effects of drought stress as well as the type of root tissues in both *Ms* and *Mt* (Supplementary Figure 2). Principal component 1 (PC1; 16.23% and 19.65% explained variance for *Ms* and *Mt*, respectively) separated tapR and fibR samples, whereas PC2 (12.69 and 13.18% for *Ms* and for *Mt*, respectively) allowed discrimination of C and drought-stressed roots (see full list of loadings in Supplementary Figure 3).

**FIGURE 2:**
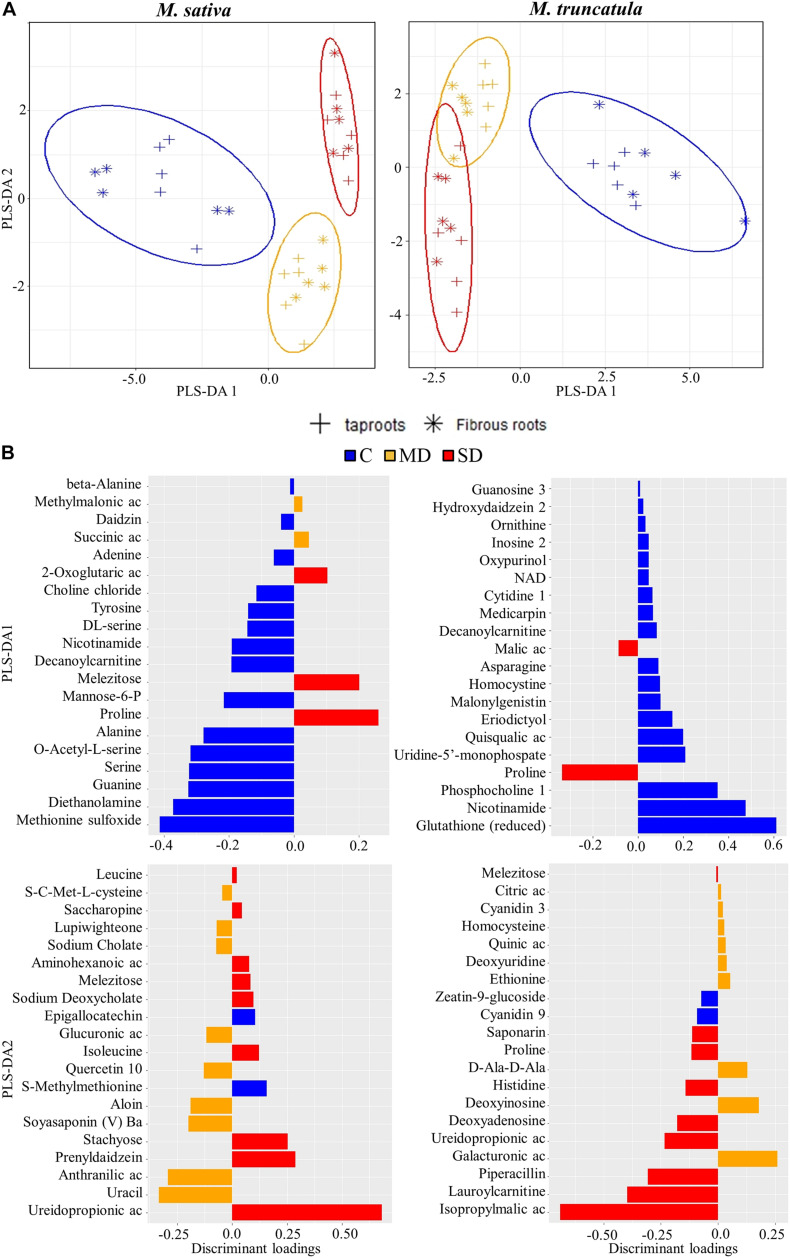
Identification of metabolites most affected by water deficit. **(A)** Partial least square discriminant analysis (PLS-DA) of metabolites in *Medicago sativa* (left panel) and *M. truncatula* (right panel). **(B)** Metabolites with the highest discriminant loadings for control (C), moderate water deficit (MD), and severe water deficit (SD) treatments. Ac, acid; NAD, nicotinamide adenine dinucleotide.

As PCA clearly separated the treatments, a subsequent PLS-DA was employed to identify the metabolites most influenced by the treatments. First, a PLS-DA was performed to identify the metabolites that responded to drought stress (Figure 2A). In both species, PLS-DA1 discriminated between C and WD-treated plant roots, whereas PLS-DA2 discriminated MD and SD root samples. Regarding PLS-DA1, the main discriminant loadings belonged to C samples in both species and included methionine sulfoxide and the reduced form of glutathione (Figure 2B). Other metabolites, such as nicotinamide, decanoylcarnitine, and numerous amino acids and organic acids also showed significantly high loadings. Proline, a classical drought marker, was found to be the most relevant metabolite changing under SD conditions in both species (Figure 2B). Regarding PLS-DA2, we identified several secondary metabolites that discriminated MD and SD, such as ureidopropionic acid, isopropylmalic acid, lauroylcarnitine, and piperacillin (Figure 2B). Regarding metabolites characterizing MD stress, we identified uracil, anthranilic acid, soyasaponin, and aloin in *Ms*, whereas galacturonic acid and deoxyinosine exhibited the highest loadings for this treatment in *Mt* (Figure 2B).

**FIGURE 3:**
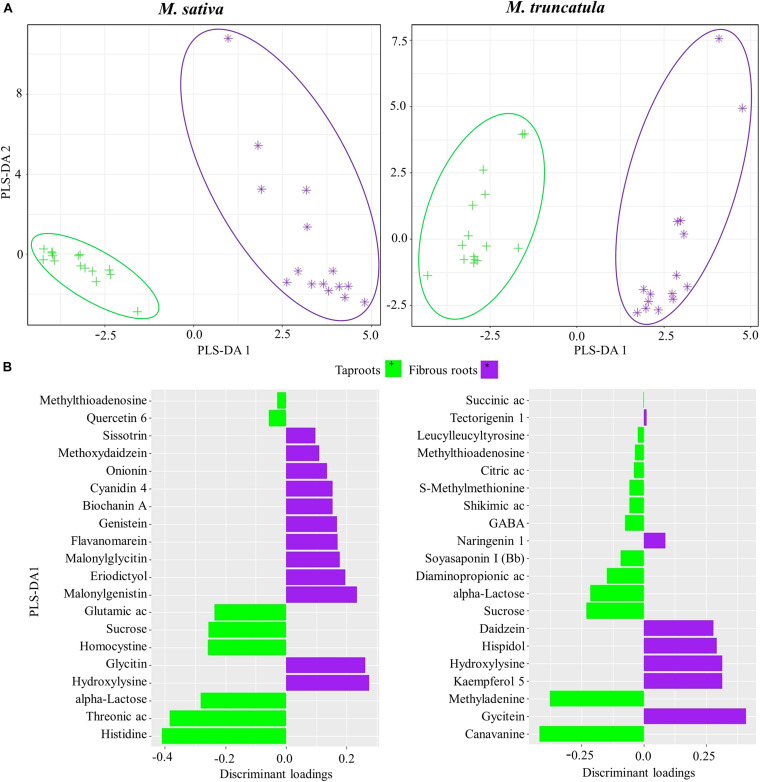
Identification of metabolites discriminating root system types. **(A)** Partial least square discriminant analysis (PLS-DA) of metabolites for *Medicago sativa* (left panel) and *M. truncatula* (right panel). **(B)** Metabolites with the highest discriminant loadings for taproots (tapRs) and fibrous roots (fibRs). Ac, acid; GABA, γ-aminobutyric acid.

Second, PLS-DA was also performed to identify the metabolites defining tapRs or fibRs in *Ms* and *Mt* species regardless of stress response (Figure 3A). We found that PLS-DA1 separated samples corresponding to different root tissues. Histidine and threonic acid were found to be candidate metabolites useful for the discrimination of tapRs in *Ms*, whereas canavanine and methyladenine showed the highest loadings in *Mt* (Figure 3B). In both species, carbohydrates, such as sucrose and alpha-lactose, were characteristic of tapRs (Figure 3B). Glycitin and glycitein were the most relevant metabolites in *Ms* and *Mt* fibRs, respectively, together with the amino acid hydroxylysine in the same tissues of both *Ms* and *Mt*. Furthermore, several flavonoids were identified as fibR-specific in both plant species (Figure 3B).

### Differential Modulation of Carbohydrate, Amino Acid, and Secondary Metabolite Levels in Drought-Stressed Plants

**FIGURE 4:**
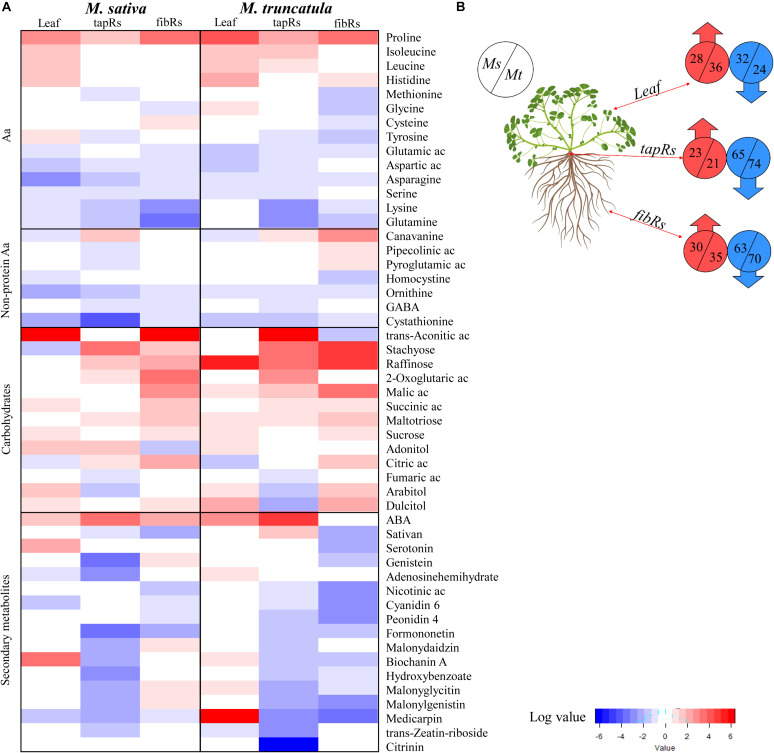
Overview of the main metabolomic changes observed under water deficit conditions. **(A)** Heatmap representing metabolites whose content was significantly altered by water deficit (Student’s *t*-test; *P* ≤ 0.05) in leaves, taproots (tapRs), and fibrous roots (fibRs) of *Medicago sativa* (*Ms*) and *M. truncatula* (*Mt*). Values were expressed as log-transformed severe water deficit/control ratios (SD/C). Cut-offs were set to two-fold change for the amino acids and carbohydrates/organic acids, and ten-fold change for secondary metabolites. **(B)** Schematic representation of the number of metabolites showing a two-fold variation in leaves, tapRs, and fibRs of *Ms* and *Mt* plants under severe water deficit (SD) conditions. ABA, abscisic acid; Ac, acid; GABA, γ-aminobutyric acid. Plant image was created with BioRender.com.

From the 287 differentially accumulated metabolites, we selected those showing significant drought-related variations based on their SD/C ratios across the different tissues. This subset was classified into five groups: proteinogenic amino acids, non-proteinogenic amino acids, carbohydrates, secondary metabolites, and miscellaneous, and their log-transformed ratios were represented as a heatmap (Figure 4A). Although the comparative analysis mostly focused on the root system, we also included metabolites present in leaves for an overview considering the whole plant. The numbers of metabolites showing significant variations in each tissue and plant species were shown in Figure 4B. Regarding amino acids, proline was systematically accumulated in all the examined organs, while leucine, isoleucine, and histidine were preferentially accumulated in leaves (Figure 4A). As carbohydrates, including sugars, organic acids, and sugar alcohols, showed the highest accumulation trends (Figure 4A), they were analyzed in further detail. Regarding secondary metabolites, a general decrease was observed in drought-stressed samples for both root types in the analyzed species (Figure 4A) with the exception of abscisic acid (ABA) that specifically accumulated in leaves and tapRs of *Mt* (Figure 4).

**FIGURE 5:**
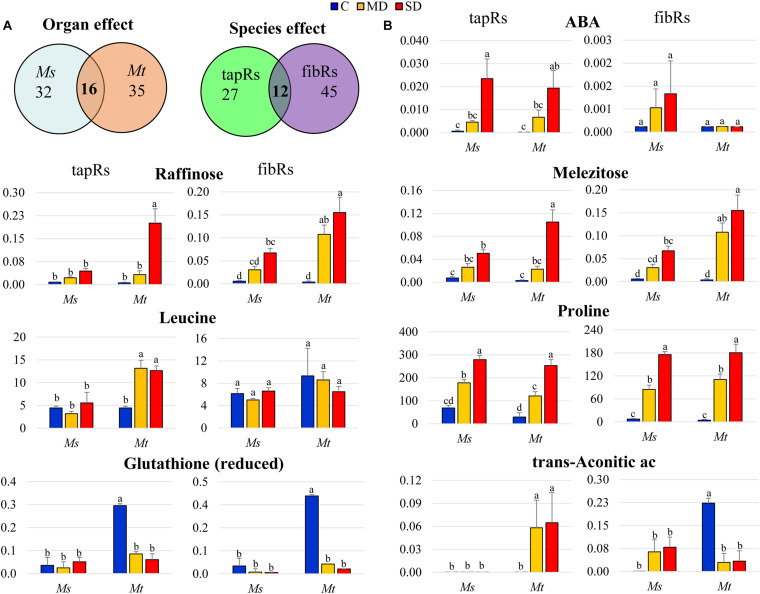
Detailed analysis of metabolites showing tissue- or stress-dependent responses. **(A)** Venn diagrams representing metabolites showing interaction between organs (left) and/or species (right) and the water deficit treatment (two-way ANOVA). **(B)** Relative intensity values of metabolites showing organ and/or species-dependent interactions with the water deficit treatment. Bars represent means ± SEs (*n* = 5 biological replicates). Different letters indicate significant differences according to a Duncan-test (*P* ≤ 0.05). Ac, acid; fibRs, fibrous roots; tapRs, taproots; *Ms*, *Medicago sativa*; *Mt*, *M. truncatula*.

The set of metabolites that were significantly changed upon drought was subjected to further analysis via two-factorial ANOVA to identify significant interactions between the factors (Figure 5A and Supplementary Table 6). A significant interaction between WD and root type was detected for approximately 50 metabolites in each species, with 16 of them being common for both *Ms* and *Mt*. Similarly, the number of metabolites showing a significant interaction between WD and species was higher in fibRs (57) than in tapRs (39), with 12 being common to both root types (Figure 5A). Regarding metabolites showing a significant interaction between root type and WD, ABA showed the clearest response, exhibiting a marked drought-related accumulation in tapRs of both species, while its content remained low in *Mt* fibRs (Figure 5B). Interestingly, raffinose showed a significant WD-organ interaction in *Ms*, exhibiting a sharp accumulation in fibRs under MD (Figure 5B). Indeed, raffinose accumulation in leaves was significantly higher in *Mt* than in *Ms* (Figure 4A). Contrastingly, reduced glutathione and *trans*-aconitic acid displayed a significant organ-WD interaction in *Mt*, with the latter showing an accumulation tapRs but a decline in *Mt* fibRs under stress conditions (Figure 5B). Concerning leaves, *trans*-aconitic acid was significantly accumulated in *Ms* (Supplementary Table 2). Regarding metabolites with a significant interaction between species and WD, raffinose and meletizose sharply accumulated in *Mt* tapRs under SD in both species (Figure 5B). Among amino acids, the branched-chain amino acid, leucine, displayed a significant species-WD interaction in tapRs, exhibiting a sharp accumulation in *Mt* while being unaffected in *Ms* (Figure 5B). Finally, proline showed a consistent accumulation in both root types of the two species, but it was remarkably accumulated in the fibRs of both *Ms* and *Mt* plants (Figure 5B) and leaves of *Mt* (Figure 4A).

### Sucrose Catabolism Displayed a Pivotal Role in the Root Response to Drought

**FIGURE 6:**
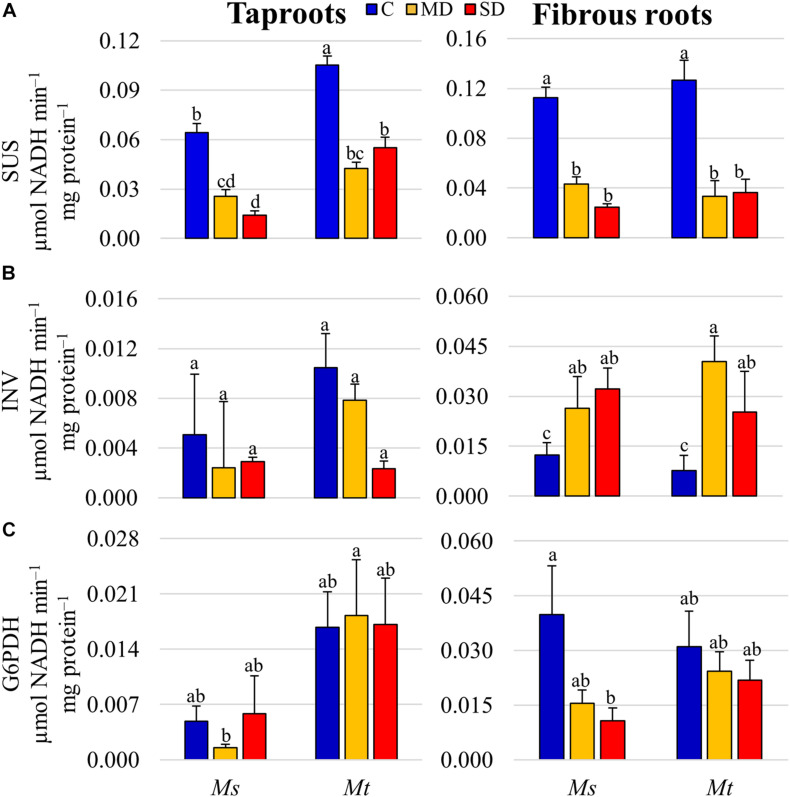
Enzymatic activities of key carbon metabolism proteins in different root tissues. **(A)** Levels of sucrose synthase (SUS), **(B)** alkaline invertase (INV), and **(C)** glucose 6-phosphate dehydrogenase (G6PDH) specific activities. Bars represent means ± SEs (*n* = 4 biological replicates). Different letters indicate significant differences according to a Duncan-test (*P* ≤ 0.05). C, control; MD, moderate water deficit; SD, severe water deficit; fibRs, fibrous roots; tapRs, taproots; *Ms, Medicago sativa; Mt, M. truncatula*.

To integrate previous data and obtain a better overview of changes in carbon metabolism, the relative variations in compounds related to carbon metabolic pathways in the different SD samples were analyzed in detail (Supplementary Figure 4 and Supplementary Table 7). We observed a general accumulation of sucrose and raffinose in all tissue samples. Interestingly, none of the sucrose degradation products were found to be differentially accumulated, suggesting that sucrose was either not degraded or metabolized to alternative carbon compounds in drought-stressed roots. This observation prompted us to further analyze the activities of enzymes and the expression of related genes involved in sucrose degradation. Thus, we measured the enzymatic activities of both SUS and INV in tapRs and fibRs in both species. SUS was the main sucrose-degrading enzyme in roots, with an average specific activity being more than 10 times larger than that of INV (Figures 6A,B). Interestingly, drought provoked a significant decline in SUS activities in both tapRs and fibRs regardless of species, and a small increase in INV activity in fibRs of both species under WD stress. In contrast, G6PDH activity, the key enzyme in the pentose phosphate pathway, only showed significant differences in *Ms* fibR (Figure 6C).

**FIGURE 7:**
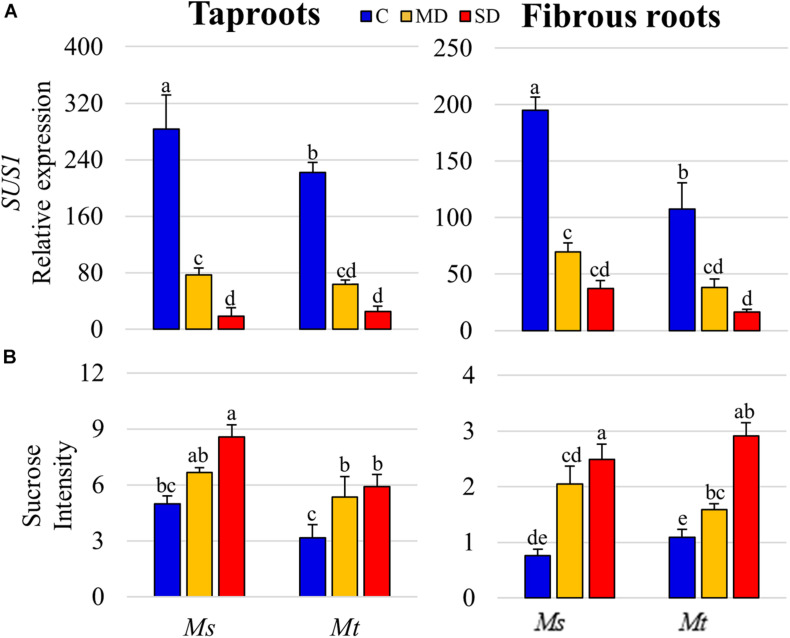
Sucrose synthase 1 (*SUS1*) gene expression and sucrose levels. **(A)** (*SUS1*, gene ID Medtr4g124660) relative gene expression and **(B)** sucrose relative levels in roots of plants subjected to water deficit. Values represent means ± SEs (*n* = 4 biological replicates). Different letters indicate significant differences according to a Duncan-test (*P* ≤ 0.05). C, control; MD, moderate water deficit; SD, severe water deficit; fibRs, fibrous roots; tapRs, taproots; *Medicago. sativa, Ms; M. truncatula, Mt*.

To test whether this reduction in SUS activity was related to changes at the transcriptional level, we analyzed the expression of *SUS* genes in both *Mt* and *Ms*. As the *SUS* family has not been fully described in legume plants, we first identified the members of the family in the genome of *Mt* (v4.0) using a BLAST-protein approach, employing the well-characterized *MtSUS1* gene (*Medtr4g124660*) as a bait. To provide additional support to the identification, we performed phylogenetic analysis of the *SUS* gene family, including those from *Mt*, *Ms*, *A. thaliana, G. max*, and *O. sativa*. Our analysis identified eight genes containing a conserved SUS protein domain (NCBI conserved domain database, PLN00142 super family): *Medtr4g124660* (*SUS1*), *Medtr7g108930* (*SUS2*), *Medtr2g044070* (*SUS3*), *Medtr1g088170* (*SUS4*), *Medtr6g478000* (*SUS5a*), *Medtr6g478030* (*SUS5b*), *Medtr3g064610* (*SUS6*), and *Medtr5g076830* (*SUS7*) (Supplementary Figure 5A). Querying available *Mt* RNA-seq gene expression databases [MtSSPdb (see text footnote 6); Boschiero et al., 2020] showed *SUS1* as the main *SUS* gene expressed in *Mt* roots (Supplementary Figure 5B). Thus, using qRT-PCR, we measured the levels of *SUS1* and its orthologous gene in the recently sequenced autotetraploid *Ms* genome (*MS.gene030241*; Chen et al., 2020), in the tapRs and fibRs subjected to drought stress. We recorded a progressive decline in *SUS1* expression levels with similar pattern to that of the activity measurements in both root types and species as drought stress increased severity (Figure 7A). Thus, the accumulation of sucrose observed during drought (Figure 7B) could be attributed to a reduction in *SUS1* expression levels, and consequently, a decline in total SUS activity in roots. The sucrose concentration determined in root tissue as absolute values showed a similar pattern in previous studies (Castañeda et al., 2019), further validating the relative values obtained in the metabolomic analysis.

## Discussion

### Water Deficit Activated the Growth of tapRs in *Ms* and fibRs in *Mt*

In this work, we characterized the physiological and metabolomic responses of two related species, *Ms* and *Mt*, subjected to progressive drought. *Mt* showed a marked decline in stomatal conductance and lower transpiration compared with those in *Ms* (Supplementary Figure 1), while presenting higher average biomass values throughout the experiment (Figure 1B). The lower transpiration rate and decrease in stomata conductance in *Mt* compared with *Ms* are common responses that have been described as crucial to maintain WC levels in various drought-tolerant legumes and other crops (Silvente et al., 2012). Under WD conditions, the shoot biomass and tissue WC were similarly reduced in both species (Figures 1B,C), in agreement with previous studies (Muller et al., 2011; Soba et al., 2019). Regarding the responses of the underground organs, *Ms* showed an increase in tapR growth, whereas *Mt* presented an increase in fibRs biomass (Figure 1B). This differential growth may be related to the perennial and annual growth habits of *Ms* and *Mt*, respectively. As previously described in Roumet et al. (2006), annual species differ from perennials in several root-related traits, the former presenting higher specific root length but lower root density and smaller root diameter. Additionally, *Ms* tapRs are adapted to penetrate the soil depth to access water resources (Humphries and Auricht, 2001; de Smet et al., 2012; Araújo et al., 2015; Huang et al., 2018), whereas fibRs are less developed than tapRs in this species (Figures 1A,B). In contrast, in *Mt*, tapRs are thinner and shorter, whereas the fibR system is largely developed (Schultz et al., 2010; Bourion et al., 2014; Castañeda et al., 2019; Figure 1A), which is a strategy to efficiently adapt to scarce precipitation in Mediterranean semi-arid environments (Wasson et al., 2012; Zhang et al., 2014).

### Drought Stress Provoked the Specific Accumulation of Metabolites in tapRs vs. fibRs

Comprehensive metabolomic analysis led us to identify specific metabolites that were differentially accumulated in plants subjected to WD conditions. This was the case of the hormone ABA, identified as a tapR-related drought marker for both *Ms* and *Mt* (Figure 5B), and considered a stress-related hormone regulating primary and lateral root growth (Sah et al., 2016; Li et al., 2017; Vishwakarma et al., 2017). A similar trend was observed for proline (Figure 5B), whose biosynthesis under drought stress is controlled by ABA (Sripinyowanich et al., 2013; Planchet et al., 2014). Indeed, proline and ABA were also found accumulated in leaves of both species (Figure 4).

In contrast, the levels of *trans*-aconitic acid, another growth-related compound typically found in forage grasses (Burau and Stout, 1965), increased in drought-stressed *Mt* tapRs but not in fibRs (Figure 5B). It would be interesting to test if this metabolite was actually responsible for the differential growth observed in these two types of roots in *Mt*, as suggested in other plant species (Voll et al., 2010; Foletto et al., 2012). At the leaf level, this organic acid was markedly accumulated in *Ms* (Supplementary Table 2). Interestingly, *trans*-aconitic acid has been related to contribute to the maintenance of redox status and energy balance in legumes (Igamberdiev and Eprintsev, 2016).

Additionally, the accumulation of several secondary metabolites related to flavonoid metabolism was associated with the drought responses in fibRs of both *Medicago* species (Figures 2, 4A). The activation of this pathway has also been described in other plant species subjected to drought (Nakabayashi et al., 2014), including some forage legumes (Ballizany et al., 2012). Interestingly, in alfalfa, drought tolerance was related to the miR156-mediated regulation of root flavonoid biosynthesis (Feyissa et al., 2019). More recently, Li et al. (2021) observed the induction of the flavonoid pathway in a drought-tolerant maize line, suggesting that flavonoids may contribute to reduce the oxidative damage and regulate stomatal movement. Thus, enhanced flavonoid biosynthesis appears to be a clear target for plant breeding strategies.

### *Ms* and *Mt* Roots Differentially Modulated a Set of Raffinose Family Oligosaccharides Under Drought

Understanding the function of abiotic stress-responsive metabolites is crucial to improve crop yield under environmental stress (Abdelrahman et al., 2018a). In particular, carbon source-sink relations are highly affected during WD stress (Rodrigues et al., 2019). Carbohydrate accumulation under WD conditions has been widely described in the leaves (Kim et al., 2000; Teulat et al., 2001) and roots (Sharp et al., 1990; Zhang et al., 2014) of different plant species, including those of the *Medicago* genus (Larrainzar et al., 2009; Kang et al., 2011; Aranjuelo et al., 2013; Castañeda et al., 2019; Molero et al., 2019). Accordingly, in this study, the levels of various carbohydrates, including oligosaccharides, polyols, and organic acids, significantly increased under drought stress at the root system level in both species (Figures 4A, 5, 7B). We detected a significant accumulation of metabolites related to the raffinose family oligosaccharide group in plants subjected to WD (Figure 5 and Supplementary Figure 4), i.e., raffinose, melezitose, and stachyose. Raffinose accumulation was significantly higher in *Mt* than in *Ms* roots under MD conditions (Figure 5). Furthermore, in leaf tissues, raffinose was found differentially accumulated in *Mt* plants only (Figure 4 and Supplementary Table 6). Similarly, melezitose showed a significant accumulation in both types of roots in *Mt*, whereas its levels remained unchanged in *Ms* roots (Figure 5). In contrast, stachyose accumulated in the roots of both species, although *Mt* roots showed an increase relatively higher than that of *Ms*, especially in fibRs (Supplementary Figure 4). These metabolites have been found to accumulate in several plant species under WD conditions (Martinelli et al., 2013; Gechev et al., 2014; Harb et al., 2015), and have even been suggested to correlate with drought tolerance when comparing different *Ms* varieties (Kang et al., 2011). Therefore, the different drought-induced accumulation of raffinose, melezitose, and stachyose between *Mt* and *Ms* in this study indicated that these are stress-responsive metabolites likely contributing to the higher drought tolerance of *Mt* compared to that of *Ms*.

### Integrative Analysis of Sucrose Catabolism Under Drought Stress Showed Regulation at Both Transcriptional and Activity Levels

Sucrose, the main end product of photosynthesis, is translocated through the phloem from source leaves to sink organs to sustain respiration and root growth (Lambers et al., 1996). Under drought, sucrose accumulation has been reported in a number of plant species (Kim et al., 2000; Hasibeder et al., 2015), including *Ms* (Kang et al., 2011; Aranjuelo et al., 2013; Molero et al., 2019) and *Mt* (Zhang et al., 2014; Castañeda et al., 2019), suggesting that sucrose may have an osmoregulatory role under WD conditions.

In sink tissues, sucrose can be either cleaved by SUS into UDP-glucose and fructose, or hydrolyzed by INV enzymes into glucose and fructose (Barratt et al., 2009). In legume root nodules exposed to water restriction, sucrose catabolism is blocked at the SUS level, which is considered a strategy to limit carbon supply to the microsymbiont under stressful conditions (Gordon et al., 1997). Subsequent studies confirmed the key role of SUS in the drought responses of several legume species in both symbiotic root nodules (Larrainzar et al., 2007, 2009; Gil-Quintana et al., 2013) and roots (Castañeda et al., 2019).

In this study, we undertook an integrative approach to further understand the role of sucrose and SUS in plant drought responses by analyzing gene expression, enzymatic activity, and metabolic levels. Enzymatic activity measurements showed that SUS is the main sucrose-cleaving enzyme in both fibRs and tapRs in *Medicago* plants. In contrast to previous studies (Xu et al., 2019), we identified 8 *SUS* genes in the *Mt* genome using a combination of reciprocal protein BLASTs and phylogenetic analysis. Among these, we quantified the expression levels of *SUS1*, the most highly expressed gene in the roots (Supplementary Figure 5B), and found a progressive drought-induced decline, particularly in tapRs (Figure 7A). This decline at the transcriptional level correlated with the reduction of SUS activity and consequent accumulation of sucrose in roots (Figures 6A, 7B). Thus, SUS activity is controlled at the transcriptional level, although post-translational mechanisms have also been suggested (Wienkoop et al., 2008 and references therein, and recently reviewed in Stein and Granot, 2019). Interestingly, INV activity showed an organ-specific response, with a significant increase in drought-stressed fibRs (Figure 6B), which agreed with the results of previous studies (Castañeda et al., 2019). This compensation of SUS and INV activities has also been reported in *Arabidopsis*, where cytosolic INV could compensate for the loss of SUS activity (Barratt et al., 2009).

## Conclusion

Physiological and metabolomic analyses allowed us to identify two different strategies to maintain plant growth and respond to WD conditions. Under drought, only tapR biomass showed a progressive increase in *Ms*, while only the fibR system showed a response in *Mt*. Interestingly, these two root systems showed contrasting metabolic compositions in response to drought, with the organ-dependent accumulation of ABA and flavonoids in tapRs and fibRs, respectively, and a remarkable accumulation of raffinose in *Mt*. Additionally, results further support the role of SUS as the main sucrose-cleaving enzyme in roots and demonstrated that the observed decline in SUS activities in drought-stressed plants corresponded to a decline in its transcript level. It will be interesting to analyze if the observed content changes in other key metabolites involved in drought responses can also be related to changes in the root transcriptome and how they may contribute to a higher drought tolerance in alfalfa at the field level. The current work provides useful information about the differential metabolic changes occurring in roots in two relevant legume species. This information provides the foundation for metabolic targeting in breeding programs and, ultimately, the development of plants with improved drought tolerance.

## Data Availability Statement

The original contributions presented in the study are included in the article/Supplementary Material, further inquiries can be directed to the corresponding author/s.

## Author Contributions

AE and EG initiated the work and created a work plan. AE performed drought stress experiments, metabolite extraction, and data analysis. WL, MS, YS, MYH, and L-SPT contributed to the planning, measurement, and analysis of metabolites. AE, YW, CT, and EL performed the qRT-PCR analysis. JM and AE performed statistical analysis. AE and EL analyzed the results and wrote the manuscript. L-SPT and EG provided edits and supervision. All authors contributed to the article and approved the submitted version.

## Conflict of Interest

The authors declare that the research was conducted in the absence of any commercial or financial relationships that could be construed as a potential conflict of interest.

## Footnotes

1 https://www.reifycs.com/AbfConverter/

2 http://prime.psc.riken.jp/compms/mrmprobs/download/repository/

3 https://phytozome.jgi.doe.gov/pz/portal.html

4 https://phytozome.jgi.doe.gov/pz/portal.html#!search?show=BLAST

5 https://www.ebi.ac.uk/Tools/phylogeny/

6 https://mtsspdb.noble.org/database/

7 https://www.genome.jp/kegg/

## Supplementary Material

Supplementary Figure 1 **(A)** Stomatal conductance and **(B)** transpiration rates in *Medicago sativa* (*Ms*) and *M. truncatula* (*Mt*) plants under control (C) and water deficit (WD) conditions during the first 7 days of treatment. Bars represent means ± SEs (*n* = 7 biological replicates). Different letters indicate significant differences according to a Duncan-test (*P* ≤ 0.05).

Supplementary Figure 2 Principal component analysis (PCA) of metabolites the root tissue of *Medicago sativa* (*Ms*) and *M. truncatula* (*Mt*) subjected to water deficit.

Supplementary Figure 3 Loading values corresponding to principal component analysis (PCA) of metabolites discriminating the effect of the root system and water deficit in *Medicago sativa* (*Ms*) and *M. truncatula* (*Mt*) plants, Ac, acid.

Supplementary Figure 4 Overview of the main metabolic pathways affected under severe water deficit (SD) conditions in *Medicago sativa* (*Ms*) and *M. truncatula* (*Mt*) in root tissue. Values represented fold-change ratios between SD and control (C) conditions. Significant increase and decrease in the content of a certain metabolite was represented in red and blue, respectively. Metabolites written in *gray* were not identified in the GC-TOF/MS-based analysis. Ac, acid; GABA, γ-aminobutyric acid; TCA, tricarboxylic acid; UDP, uridine diphosphate; fibrous roots, fibRs; taproots, tapRs.

Supplementary Figure 5 **(A)** Phylogenetic analysis of the sucrose synthase (SUS) family in *Medicago truncatula* (*Mt*), *Oryza sativa*, *Glycine max*, and *Arabidopsis thaliana*. A maximum likelihood tree was constructed with MEGA 10.1 (https://www.megasoftware.net/) using the predicted amino acid sequences based on the Whelan And Goldman (WAG) model (Whelan and Goldman, 2001). Values on the branches represented bootstrap values (1,000 repetitions). *SUS* gene names for *Mt* and *A. thaliana* were specified in brackets. **(B)** Expression levels of the SUS family in *Mt* based on the RNA-sequencing data extracted from the MtSSPdb database (https://mtsspdb.noble.org/database/). RPM, reads per million.

Supplementary Table 1 Metabolomic analysis methods and matrices. Complete list of identified metabolites, parameters for the separation and mass spectrometric identification of compounds, and raw and normalized matrices of quantified metabolites.

Supplementary Table 2 Relative quantification and fold change ratios of metabolites detected in *Medicago sativa* and *M. truncatula* leaf, taproot and fibrous root tissues under control (C), moderate deficit (MD), and severe deficit (SD) conditions. Values represent means ± SEs (*n* = 5 biological replicates), Ac, acid.

Supplementary Table 3 Primer sequences used for qRT-PCR analysis. *Ms*, *Medicago sativa*; *Mt*, *M. truncatula*; *SUS1*, sucrose synthase 1.

Supplementary Table 4 Interactions between plant species/organ and treatments using two-way ANOVA statistical analysis. ^∗∗∗^, ^∗∗^, and ^∗^ asterisks indicate statistical significance at the 0.001, 0.01, and 0.05 level, respectively; ns, not significant at the 0.05 level. C, carbon; DW, dry weight; G6PDH, glucose-6-phosphate dehydrogenase; INV, alkaline invertase; N, nitrogen; SUS, sucrose synthase; WD, water deficit.

Supplementary Table 5 List of metabolites with contents significantly altered by severe water deficit (Student’s *t*-test; *P* ≤ 0.05) in leaves, taproots (tapRs), and fibrous roots (fibRs) of *Medicago sativa (Ms)* and *M. truncatula* (*Mt*). Metabolites identified in Figures 3 and 4 with a discriminant loading value higher than 0.2 were marked with gray background and represented in the heatmap (Figure 5) with bold letters. ID represent each metabolite identifier in the analysis extracted from Supplementary Table 1.

Supplementary Table 6 List of metabolites showing an organ or species interaction based on ANOVA, as represented in Figure 5. Values represent means ± SEs (*n* = 5 biological replicates) of normalized intensity values. Metabolites showing organ effects in *Medicago sativa, M. truncatula*, and both species were represented in green, orange, and brown, respectively. Metabolites showing species-related effects in taproots, fibrous roots and both root systems were represented in yellow, blue, and gray, respectively. Metabolites sharing organ effects to both species and species effects in both organs were represented in bold. C, control; MD, moderate water deficit; SD, severe water deficit; fibRs, fibrous roots; tapRs, taproots; *Ms, Medicago sativa; Mt, M. truncatula*.

Supplementary Table 7 Normalized values of metabolites related to carbon metabolism detected in taproots (tapRs) and fibrous roots (fibRs) of *Medicago sativa* (*Ms*) and *M truncatula* (*Mt*) plants under control (C) and severe deficit (SD) conditions. Values highlighted in orange indicate a significant increase in SD plants relative to well-irrigated plants (C). Values highlighted in blue indicate a significant decrease in tissues subjected to water deficit relative to C plants. Data were presented as means ± SEs (*n* = 5 biological replicates). Different letters indicate significant differences as determined by a Duncan test (*P* ≤ 0.05). Ac, acid; GABA, γ-aminobutyric acid.

## Abbreviations

ABA: abscisic acid
Asn: asparagine
C: control
DW: dry weight
fibRs: fibrous roots
FW: fresh weight
GABA: γ-aminobutyric acid
INV: alkaline invertase
LC-QqQ-MS: liquid chromatography with triple-quadrupole mass spectrometry
MD: moderate water deficit
SD: severe water deficit
SUS: sucrose synthase
*SUS*: sucrose synthase gene family
tapRs: taproots
WC: water content
WD: water deficit.

## References

[B1] Abdelrahman M. Burritt D. J. Tran L. S. P. (2018a). The use of metabolomic quantitative trait locus mapping and osmotic adjustment traits for the improvement of crop yields under environmental stresses. *Semin. Cell Dev. Biol.* 83 86–94. 10.1016/j.semcdb.2017.06.020 28668354

[B2] Abdelrahman M. Jogaiah S. Burritt D. J. Tran L. S. P. (2018b). Legume genetic resources and transcriptome dynamics under abiotic stress conditions. *Plant Cell Environ.* 41 1972–1983. 10.1111/pce.13123 29314055

[B3] Anjum S. A. Ashraf U. Zohaib A. Tanveer M. Naeem M. Ali I. (2017). Growth and developmental responses of crop plants under drought stress: a review. *Zemdirbyste* 104 267–276. 10.13080/z-a.2017.104.034

[B4] Annicchiarico P. Barrett B. Brummer E. C. Julier B. Marshall A. H. (2015). Achievements and challenges in improving temperate perennial forage legumes. *CRC Crit. Rev. Plant Sci.* 34 327–380. 10.1080/07352689.2014.898462

[B5] Annicchiarico P. Pecetti L. Abdelguerfi A. Bouizgaren A. Carroni A. M. Hayek T. (2011). Adaptation of landrace and variety germplasm and selection strategies for lucerne in the Mediterranean basin. *Field Crop. Res.* 120 283–291. 10.1016/j.fcr.2010.11.003

[B6] Aranjuelo I. Tcherkez G. Molero G. Gilard F. Avice J.-C. Nogués S. (2013). Concerted changes in N and C primary metabolism in alfalfa (*Medicago sativa*) under water restriction. *J. Exp. Bot.* 64 1–17. 10.1093/jxb/ers367 23440170 PMC3580806

[B7] Araújo S. S. Beebe S. Crespi M. Delbreil B. González E. M. Gruber V. (2015). Abiotic stress responses in legumes: strategies used to cope with environmental challenges. *CRC Crit. Rev. Plant Sci.* 34 237–280. 10.1080/07352689.2014.898450

[B8] Aubert G. Morin J. Jacquin F. Loridon K. Quillet M. C. Petit A. (2006). Functional mapping in pea, as an aid to the candidate gene selection and for investigating synteny with the model legume *Medicago truncatula*. *Theor. Appl. Genet.* 112 1024–1041. 10.1007/s00122-005-0205-y 16416153

[B9] Ballizany W. L. Hofmann R. W. Jahufer M. Z. Z. Barrett B. A. (2012). Multivariate associations of flavonoid and biomass accumulation in white clover (*Trifolium repens*) under drought. *Funct. Plant Biol.* 39 167–177. 10.1071/FP11193 32480771

[B10] Barker D. G. Bianchl S. Blondon F. Duc G. Essad S. Flament P. (1990). *Medicago truncatula*, a model plant for studying the molecular genetics of the Rhizobium-legume symbiosis. *Plant Mol. Biol. Rep.* 8 40–49. 10.1007/BF02668879

[B11] Barratt D. H. P. Derbyshire P. Findlay K. Pike M. Wellner N. Lunn J. (2009). Normal growth of *Arabidopsis* requires cytosolic invertase but not sucrose synthase. *PNAS* 106 13124–13129. 10.1073/pnas.0900689106 19470642 PMC2722301

[B12] Boschiero C. Dai X. Lundquist P. K. Roy S. de Bang T. C. Zhang S. (2020). MtSSPDB: the *Medicago truncatula* small secreted peptide database. *Plant Physiol.* 183 399–413. 10.1104/pp.19.01088 32079733 PMC7210635

[B13] Bourion V. Martin C. De Larambergue H. Jacquin F. Aubert G. Martin-Magniette M. L. (2014). Unexpectedly low nitrogen acquisition and absence of root architecture adaptation to nitrate supply in a *Medicago truncatula* highly branched root mutant. *J. Exp. Bot.* 65 2365–2380. 10.1093/jxb/eru124 24706718 PMC4036509

[B14] Bradford M. M. (1976). A rapid and sensitive method for the quantitation of microgram quantities of protein utilizing the principle of protein-dye binding. *Anal. Biochem.* 72 248–254. 10.1016/0003-2697(76)90527-3 942051

[B15] Burau R. Stout P. R. (1965). Trans-Aconitic acid in range grasses in early spring. *Science* 150 766–767. 10.1126/science.150.3697.766 17835250

[B16] Castañeda V. de la Peña M. Azcárate L. Aranjuelo I. Gonzalez E. M. (2019). Functional analysis of the taproot and fibrous roots of *Medicago truncatula*: sucrose and proline catabolism primary response to water deficit. *Agric. Water Manag.* 216 473–483. 10.1016/j.agwat.2018.07.018

[B17] Chen H. Zeng Y. Yang Y. Huang L. Tang B. Zhang H. (2020). Allele-aware chromosome-level genome assembly and efficient transgene-free genome editing for the autotetraploid cultivated alfalfa. *Nat. Commun.* 11:2494. 10.1038/s41467-020-16338-x 32427850 PMC7237683

[B18] Daryanto S. Wang L. Jacinthe P. A. (2015). Global synthesis of drought effects on food legume production. *PLoS One* 10:e0127401. 10.1371/journal.pone.0127401 26061704 PMC4464651

[B19] de Smet I. White P. J. Glyn Bengough A. Dupuy L. Parizot B. Casimiro I. (2012). Analyzing lateral root development: how tomove forward. *Plant Cell* 24 15–20. 10.1105/tpc.111.094292 22227890 PMC3289553

[B20] Feyissa B. A. Arshad M. Gruber M. Y. Kohalmi S. E. Hannoufa A. (2019). The interplay between *miR156/SPL13* and *DFR/WD40-1* regulate drought tolerance in alfalfa. *BMC Plant Biol.* 19:434. 10.1186/s12870-019-2059-5 31638916 PMC6802326

[B21] Foletto M. P. Kagami F. Voll E. Kern-Cardoso K. A. Pergo-Coelho E. M. Rocha M. (2012). Allelopathic effects of *Brachiaria ruziziensis* and aconitic acid on *Ipomoea triloba* weed. *Allelopath. J.* 30 33–48.

[B22] Garcia J. Barker D. J. Journet E.-P. (2006). “Seed storage and germination,” in *The Medicago truncatula Handbook. The Samuel Roberts Noble Foundation*, ed. Mathesius U. (Ardmore, OK: Noble Research Institute).

[B23] Gechev T. S. Hille J. Woerdenbag H. J. Benina M. Mehterov N. Toneva V. (2014). Natural products from resurrection plants: potential for medical applications. *Biotechnol. Adv.* 32 1091–1101. 10.1016/j.biotechadv.2014.03.005 24681091

[B24] Gil-Quintana E. Larrainzar E. Seminario A. Díaz-Leal J. L. Alamillo J. M. Pineda M. (2013). Local inhibition of nitrogen fixation and nodule metabolism in drought-stressed soybean. *J. Exp. Bot.* 64 2171–2182. 10.1093/jxb/ert074 23580751 PMC3654410

[B25] Gordon A. J. Minchin F. R. Skøt L. James C. L. (1997). Stress-induced declines in soybean N2 fixation are related to nodule sucrose synthase activity. *Plant Physiol.* 114 937–946. 10.1104/pp.114.3.937 12223754 PMC158382

[B26] Graham P. H. Vance C. P. (2003). Legumes: importance and constraints to greater use. *Plant Physiol.* 131 872–877. 10.1104/pp.017004 12644639 PMC1540286

[B27] Harb J. Alseekh S. Tohge T. Fernie A. R. (2015). Profiling of primary metabolites and flavonols in leaves of two table grape varieties collected from semiarid and temperate regions. *Phytochemistry* 117 444–455. 10.1016/j.phytochem.2015.07.013 26196939

[B28] Hasibeder R. Fuchslueger L. Richter A. Bahn M. (2015). Summer drought alters carbon allocation to roots and root respiration in mountain grassland. *New Phytol.* 205 1117–1127. 10.1111/nph.13146 25385284 PMC4303983

[B29] Hu Y. Schmidhalter U. (2005). Drought and salinity: a comparison of their effects on mineral nutrition of plants. *J. Plant Nutr. Soil Sci.* 168 541–549. 10.1002/jpln.200420516

[B30] Huang Z. Liu Y. Cui Z. Fang Y. He H. Liu B. R. (2018). Soil water storage deficit of alfalfa (*Medicago sativa*) grasslands along ages in arid area (China). *Field Crop. Res.* 221 1–6. 10.1016/j.fcr.2018.02.013

[B31] Humphries A. W. Auricht G. C. (2001). Breeding lucerne for Australia’s southern dryland cropping environments. *Aust. J. Agric. Res.* 52 153–169. 10.1071/AR99171

[B32] Igamberdiev A. U. Eprintsev A. T. (2016). Organic acids: the pools of fixed carbon involved in redox regulation and energy balance in higher plants. *Front. Plant Sci.* 7:1042. 10.3389/fpls.2016.01042 27471516 PMC4945632

[B33] Kanehisa M. Goto S. (2000). KEGG: kyoto encyclopedia of genes and genomes. *Nucleic Acids* 28 27–30. 10.1093/nar/28.1.27 PMC102409 10592173

[B34] Kanehisa M. Sato Y. Kawashima M. Furumichi M. Tanabe M. (2015). KEGG as a reference resource for gene and protein annotation. *Nucleic Acids* 44 457–462. 10.1093/nar/gkv1070 PMC4702792 26476454

[B35] Kang Y. Han Y. Torres-Jerez I. Wang M. Tang Y. Monteros M. (2011). System responses to long-term drought and re-watering of two contrasting alfalfa varieties. *Plant J.* 68 871–889. 10.1111/j.1365-313X.2011.04738.x 21838776

[B36] Kim J. Y. Mahe A. Brangeon J. Prioul J. L. (2000). A maize vacuolar invertase, *IVR2*, is induced by water stress. Organ/tissue specificity and diurnal modulation of expression. *Plant Physiol.* 124 71–84. 10.1104/pp.124.1.71 10982423 PMC59123

[B37] Kumar S. Stecher G. Li M. Knyaz C. Tamura K. (2018). MEGA X: molecular evolutionary genetics analysis across computing platforms. *Mol. Biol. Evol.* 35 1547–1549. 10.1093/molbev/msy096 29722887 PMC5967553

[B38] Lambers H. Stulen I. van der Werf A. (1996). Carbon use in root respiration as affected by elevated atmospheric CO_2_. *Plant Soil* 187 251–263. 10.1007/BF00017091

[B39] Larrainzar E. Wienkoop S. Scherling C. Kempa S. Ladrera R. Arrese-Igor C. (2009). Carbon metabolism and bacteroid functioning are involved in the regulation of nitrogen fixation in *Medicago truncatula* under drought and recovery. *Mol. Plant Microbe Interact.* 22 1565–1576. 10.1094/mpmi-22-12-1565 19888822

[B40] Larrainzar E. Wienkoop S. Weckwerth W. Ladrera R. Arrese-Igor C. González E. M. (2007). *Medicago truncatula* root nodule proteome analysis reveals differential plant and bacteroid responses to drought stress. *Plant Physiol.* 144 1495–1507. 10.1104/pp.107.101618 17545507 PMC1914115

[B41] Le D. T. Aldrich D. L. Valliyodan B. Watanabe Y. van Ha C. Nishiyama R. (2012). Evaluation of candidate reference genes for normalization of quantitative RT-PCR in soybean tissues under various abiotic stress conditions. *PLoS One* 7:e46487. 10.1371/journal.pone.0046487 23029532 PMC3460875

[B42] Li B. Fan R. Sun G. Sun T. Fan Y. Bai S. (2021). Flavonoids improve drought tolerance of maize seedlings by regulating the homeostasis of reactive oxygen species. *Plant Soil* (in press). 10.1007/s11104-020-04814-8

[B43] Li X. Chen L. Forde B. G. Davies W. J. (2017). The biphasic root growth response to abscisic acid in arabidopsis involves interaction with ethylene and auxin signalling pathways. *Front. Plant Sci.* 8:1493. 10.3389/fpls.2017.01493 28890725 PMC5574904

[B44] Martinelli F. Remorini D. Saia S. Massai R. Tonutti P. (2013). Metabolic profiling of ripe olive fruit in response to moderate water stress. *Sci. Hortic.* 159 52–58. 10.1016/j.scienta.2013.04.039

[B45] Molero G. Tcherkez G. Roca R. Mauve C. Cabrera-Bosquet L. Araus J. L. (2019). Do metabolic changes underpin physiological responses to water limitation in alfalfa (*Medicago sativa*) plants during a regrowth period? *Agric. Water Manag.* 212 1–11. 10.1016/j.agwat.2018.08.021

[B46] Muller B. Pantin F. Génard M. Turc O. Freixes S. Piques M. (2011). Water deficits uncouple growth from photosynthesis, increase C content, and modify the relationships between C and growth in sink organs. *J. Exp. Bot.* 62 1715–1729. 10.1093/jxb/erq438 21239376

[B47] Nakabayashi R. Yonekura-Sakakibara K. Urano K. Suzuki M. Yamada Y. Nishizawa T. (2014). Enhancement of oxidative and drought tolerance in Arabidopsis by overaccumulation of antioxidant flavonoids. *Plant J.* 77 367–379. 10.1111/tpj.12388 24274116 PMC4282528

[B48] Phan H. T. T. Ellwood S. R. Hane J. K. Ford R. Materne M. Oliver R. P. (2007). Extensive macrosynteny between *Medicago truncatula* and *Lens culinaris* ssp. culinaris. *Theor. Appl. Genet.* 114 549–558. 10.1007/s00122-006-0455-3 17119911

[B49] Planchet E. Verdu I. Delahaie J. Cukier C. Girard C. Morère-Le Paven M. C. (2014). Abscisic acid-induced nitric oxide and proline accumulation in independent pathways under water-deficit stress during seedling establishment in *Medicago truncatula*. *J. Exp. Bot.* 65 2161–2170. 10.1093/jxb/eru088 24604737

[B50] Quan W. Liu X. Wang H. Chan Z. (2016). Comparative physiological and transcriptional analyses of two contrasting drought tolerant alfalfa varieties. *Front. Plant Sci.* 6:1256. 10.3389/fpls.2015.01256 26793226 PMC4709457

[B51] Radovic J. Sokolovic D. Markovic J. (2009). Alfalfa-most important perennial forage legume in animal husbandry. *Biotechnol. Anim. Husb.* 25 465–475. 10.2298/bah0906465r

[B52] Ray I. M. Han Y. Lei E. Meenach C. D. Santantonio N. Sledge M. K. (2015). Identification of quantitative trait loci for alfalfa forage biomass productivity during drought stress. *Crop Sci.* 55 2012–2033. 10.2135/cropsci2014.12.0840

[B53] Rodrigues J. Inzé D. Nelissen H. Saibo N. J. M. (2019). Source–Sink regulation in crops under water deficit. *Trends Plant Sci.* 24 652–663. 10.1016/j.tplants.2019.04.005 31109763

[B54] Rohart F. Gautier B. Singh A. Lê Cao K.-A. (2017). mixOmics: an R package for ‘omics feature selection and multiple data integration. *PLoS Comput. Biol.* 13:e1005752. 10.1371/journal.pcbi.1005752 29099853 PMC5687754

[B55] Roumet C. Urcelay C. Díaz S. (2006). Suites of root traits differ between annual and perennial species growing in the field. *New Phytol.* 170, 357–368. 10.1111/j.1469-8137.2006.01667.x 16608460

[B56] Sah S. K. Reddy K. R. Li J. (2016). Abscisic acid and abiotic stress tolerance in crop plants. *Front. Plant Sci.* 7:571. 10.3389/fpls.2016.00571 27200044 PMC4855980

[B57] Sañko-Sawczenko I. Łotocka B. Mielecki J. Rekosz-Burlaga H. Czarnocka W. (2019). Transcriptomic changes in *Medicago truncatula* and *Lotus japonicus* root nodules during drought stress. *Int. J. Mol. Sci.* 20:1204. 10.3390/ijms20051204 30857310 PMC6429210

[B58] Scholander P. F. Bradstreet E. D. Hemmingsen E. A. Hammel H. T. (1965). Sap pressure in vascular plants: negative hydrostatic pressure can be measured in plants. *Science* 148 339–346. 10.1126/science.148.3668.339 17832103

[B59] Schultz C. J. Kochian L. V. Harrison M. J. (2010). Genetic variation for root architecture, nutrient uptake and mycorrhizal colonisation in *Medicago truncatula* accessions. *Plant Soil* 336 113–128. 10.1007/s11104-010-0453-8

[B60] Sharp R. E. Hsiao T. C. Silk W. K. (1990). Growth of the maize primary root at low water potentials: II. Role of growth and deposition of hexose and potassium in osmotic adjustment. *Plant Physiol.* 93 1337–1346. 10.1104/pp.93.4.1337 16667622 PMC1062677

[B61] Sheaffer C. C. Tanner C. B. Kirkham M. B. (1988). “Alfalfa water and irrigation,” in *Alfalfa and Alfalfa Improvement*, eds Hansons A. A. Barnes D. K. Hill J. R. (Madison, WI: American Society of Agronomy, Inc.), 373–409. 10.2134/agronmonogr29.c11

[B62] Silvente S. Sobolev A. P. Lara M. (2012). Metabolite adjustments in drought tolerant and sensitive soybean genotypes in response to water stress. *PLoS ONE* 7:e38554. 10.1371/journal.pone.0038554 22685583 PMC3369847

[B63] Soba D. Zhou B. Arrese-Igor C. Munné-Bosch S. Aranjuelo I. (2019). Physiological, hormonal and metabolic responses of two alfalfa cultivars with contrasting responses to drought. *Int. J. Mol. Sci.* 20:5099. 10.3390/ijms20205099 31618819 PMC6829892

[B64] Sripinyowanich S. Klomsakul P. Boonburapong B. Bangyeekhun T. Asami T. Gu H. (2013). Exogenous ABA induces salt tolerance in indica rice (*Oryza sativa* L.): the role of *OsP5CS1* and *OsP5CR* gene expression during salt stress. *Environ. Exp. Bot.* 86 94–105. 10.1016/j.envexpbot.2010.01.009

[B65] Stein O. Granot D. (2019). An overview of sucrose synthases in plants. *Front. Plant Sci.* 10:95. 10.3389/fpls.2019.00095 30800137 PMC6375876

[B66] Teulat B. Borries C. This D. (2001). New QTLs identified for plant water status, water-soluble carbohydrate and osmotic adjustment in a barley population grown in a growth-chamber under two water regimes. *Theor. Appl. Genet.* 103 161–170. 10.1007/s001220000503

[B67] Tian H. De Smet I. Ding Z. (2014). Shaping a root system: regulating lateral versus primary root growth. *Trends Plant Sci.* 19 426–431. 10.1016/j.tplants.2014.01.007 24513255

[B68] Tsugawa H. Arita M. Kanazawa M. Ogiwara A. Bamba T. Fukusaki E. (2013). MRMPROBS: a data assessment and metabolite identification tool for large-scale multiple reaction monitoring based widely targeted metabolomics. *Anal. Chem.* 85 5191–5199. 10.1021/ac400515s 23581547

[B69] Vance C. P. Graham P. H. Allan D. L. (2000). “Biological nitrogen fixation: phosphorus - a critical future need?,” in *Nitrogen Fixation: From Molecules to Crop Productivity*, eds Pedrosa O. Hungria M. Yates M. Newton W. (Dordrecht: Kluwer Academic Publishers), 509–514. 10.1007/0-306-47615-0_291

[B70] Vishwakarma K. Upadhyay N. Kumar N. Yadav G. Singh J. Mishra R. K. (2017). Abscisic acid signaling and abiotic stress tolerance in plants: a review on current knowledge and future prospects. *Front. Plant Sci.* 8:161. 10.3389/fpls.2017.00161 28265276 PMC5316533

[B71] Voll E. Gazziero D. L. P. Adegas F. S. (2010). Aconitic acid on seeds of weed species from different locations. *Planta Daninha* 28 13–22. 10.1590/S0100-83582010000100002

[B72] Wasaya A. Zhang X. Fang Q. Yan Z. (2018). Root phenotyping for drought tolerance: a review. *Agronomy* 8, 1–19. 10.3390/agronomy8110241

[B73] Wasson A. P. Richards R. A. Chatrath R. Misra S. C. Prasad S. V. S. Rebetzke G. J. (2012). Traits and selection strategies to improve root systems and water uptake in water-limited wheat crops. *J. Exp. Bot.* 63 3485–3498. 10.1093/jxb/ers111 22553286

[B74] Whelan S. Goldman N. (2001). A general empirical model of protein evolution derived from multiple protein families using a maximum-likelihood approach. *Mol. Biol. Evol.* 18 691–699. 10.1093/oxfordjournals.molbev.a003851 11319253

[B75] Wienkoop S. Larrainzar E. Glinski M. González E. M. Arrese-Igor C. Weckwerth W. (2008). Absolute quantification of *Medicago truncatula* sucrose synthase isoforms and N-metabolism enzymes in symbiotic root nodules and the detection of novel nodule phosphoproteins by mass spectrometry. *J. Exp. Bot.* 59 3307–3315. 10.1093/jxb/ern182 18772307 PMC2529246

[B76] Xu X. Yang Y. Liu C. Sun Y. Zhang T. Hou M. (2019). The evolutionary history of the sucrose synthase gene family in higher plants. *BMC Plant Biol.* 19:566. 10.1186/s12870-019-2181-4 31852440 PMC6921546

[B77] Young N. D. Udvardi M. (2009). Translating *Medicago truncatula* genomics to crop legumes. *Curr. Opin. Plant Biol.* 12 193–201. 10.1016/J.PBI.2008.11.005 19162532

[B78] Zandalinas S. I. Mittler R. Balfagón D. Arbona V. Gómez-Cadenas A. (2018). Plant adaptations to the combination of drought and high temperatures. *Physiol. Plant* 162 1–12. 10.1111/ppl.12540 28042678

[B79] Zhang C. Shi S. Liu Z. Yang F. Yin G. (2019). Drought tolerance in alfalfa (*Medicago sativa* L.) varieties is associated with enhanced antioxidative protection and declined lipid peroxidation. *J. Plant Physiol.* 232 226–240. 10.1016/J.JPLPH.2018.10.023 30537610

[B80] Zhang J. Y. Cruz, De Carvalho M. H. Torres-Jerez I. Kang Y. Allen S. N. (2014). Global reprogramming of transcription and metabolism in *Medicago truncatula* during progressive drought and after rewatering. *Plant Cell Environ.* 37 2553–2576. 10.1111/pce.12328 24661137 PMC4260174

[B81] Zhang T. Yu L.-X. Zheng P. Li Y. Rivera M. Main D. (2015). Identification of loci associated with drought resistance traits in heterozygous autotetraploid alfalfa (*Medicago sativa* L.) using genome-wide association studies with genotyping by sequencing. *PLoS One* 10:e0138931. 10.1371/journal.pone.0138931 26406473 PMC4583413

