# Supplementary figures and images for: Medicago sativa and Medicago truncatula Show Contrasting Root Metabolic Responses to Drought
Source: Front Plant Sci. 2021 Apr 21;12:652143. doi: 10.3389/fpls.2021.652143 (PMC8097159; doi:10.3389/fpls.2021.652143)

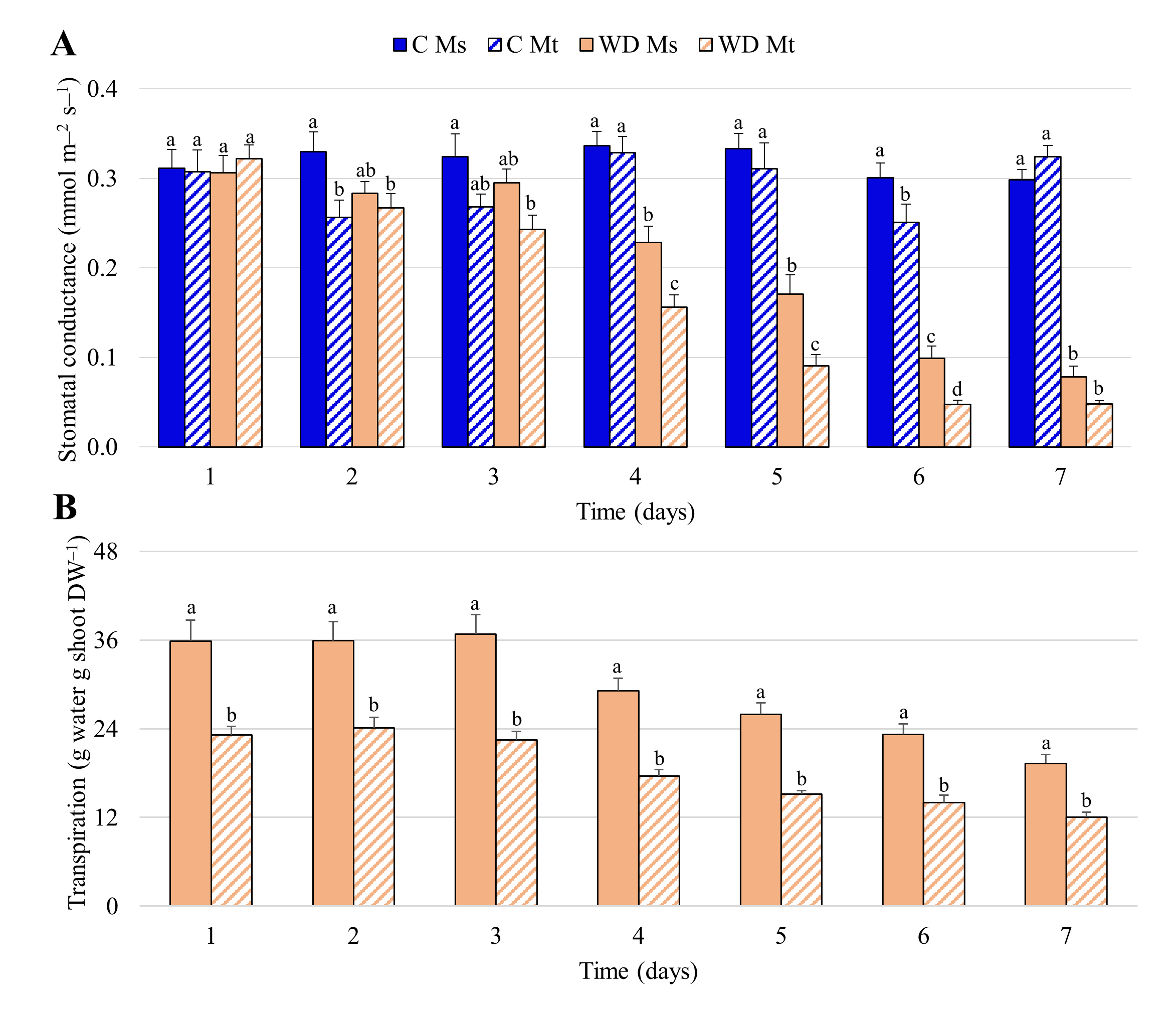

Supplement: Supplementary Figure 1 — (A) Stomatal conductance and (B) transpiration rates in Medicago sativa (Ms) and M. truncatula (Mt) plants under control (C) and water deficit (WD) conditions during the first 7 days of treatment. Bars represent means ± SEs (n = 7 biological replicates). Different letters indicate significant differences according to a Duncan-test (P ≤ 0.05). [file Image_1.TIF]

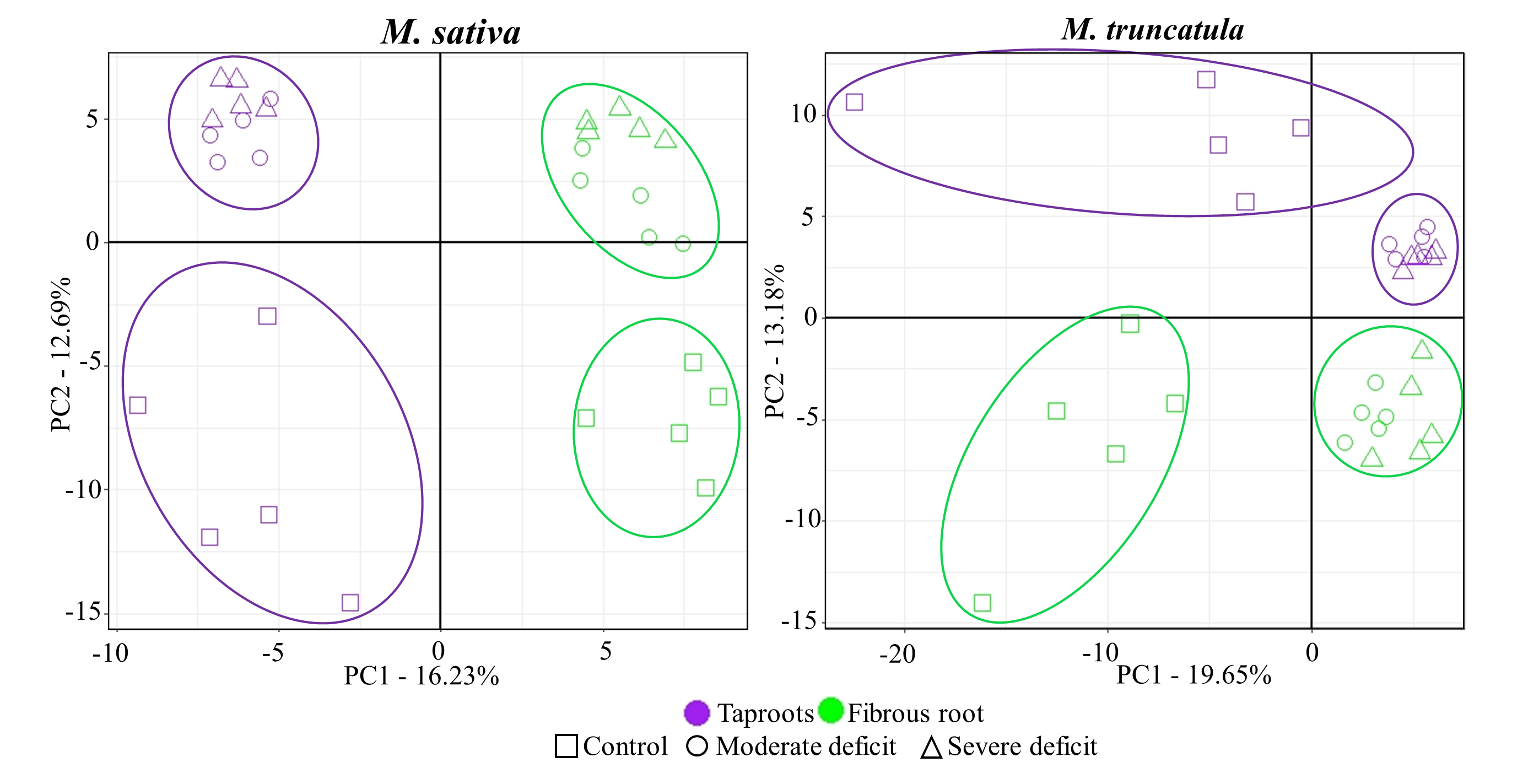

Supplement: Supplementary Figure 2 — Principal component analysis (PCA) of metabolites the root tissue of Medicago sativa (Ms) and M. truncatula (Mt) subjected to water deficit. [file Image_2.TIF]

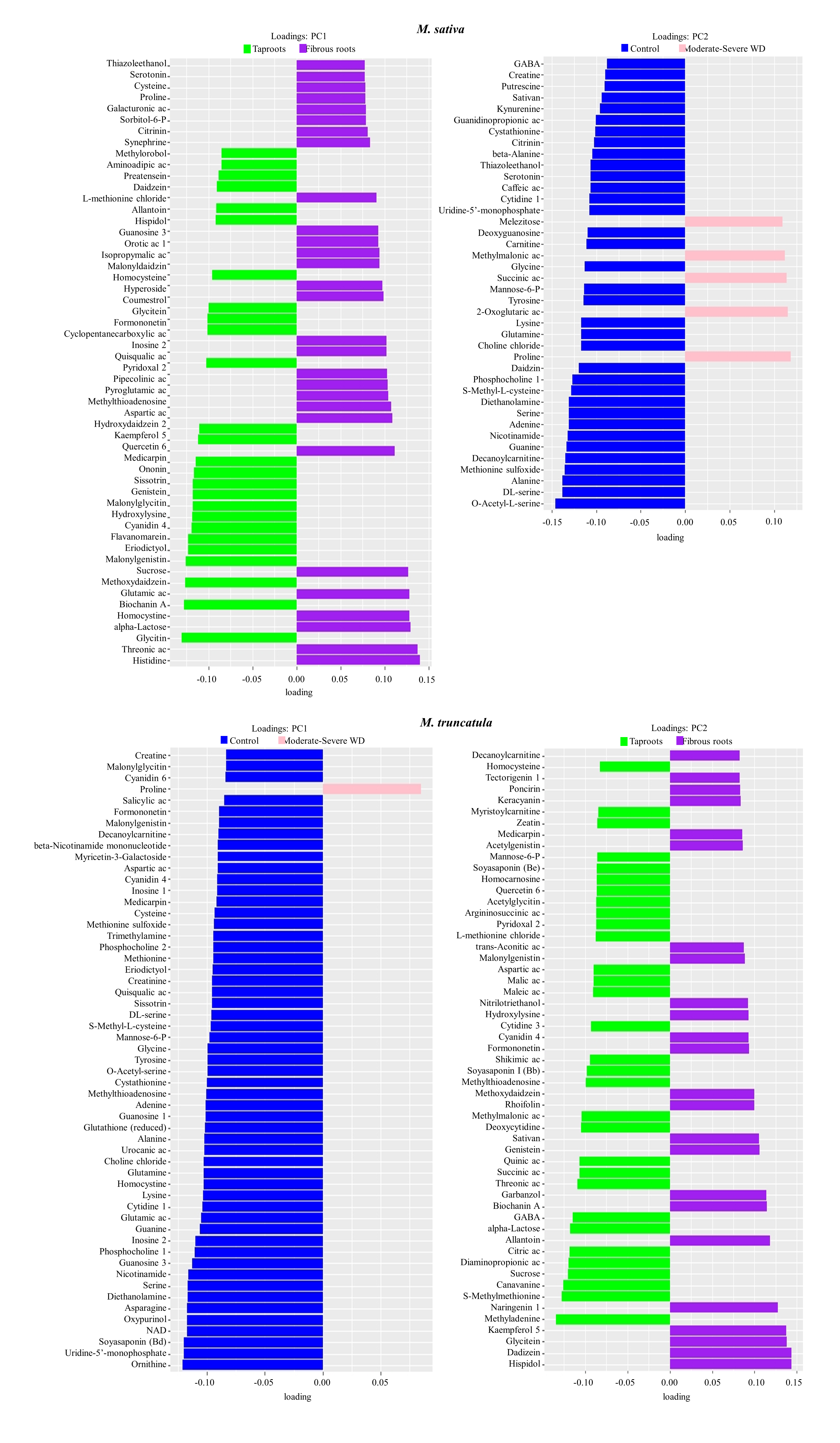

Supplement: Supplementary Figure 3 — Loading values corresponding to principal component analysis (PCA) of metabolites discriminating the effect of the root system and water deficit in Medicago sativa (Ms) and M. truncatula (Mt) plants, Ac, acid. [file Image_3.TIF]

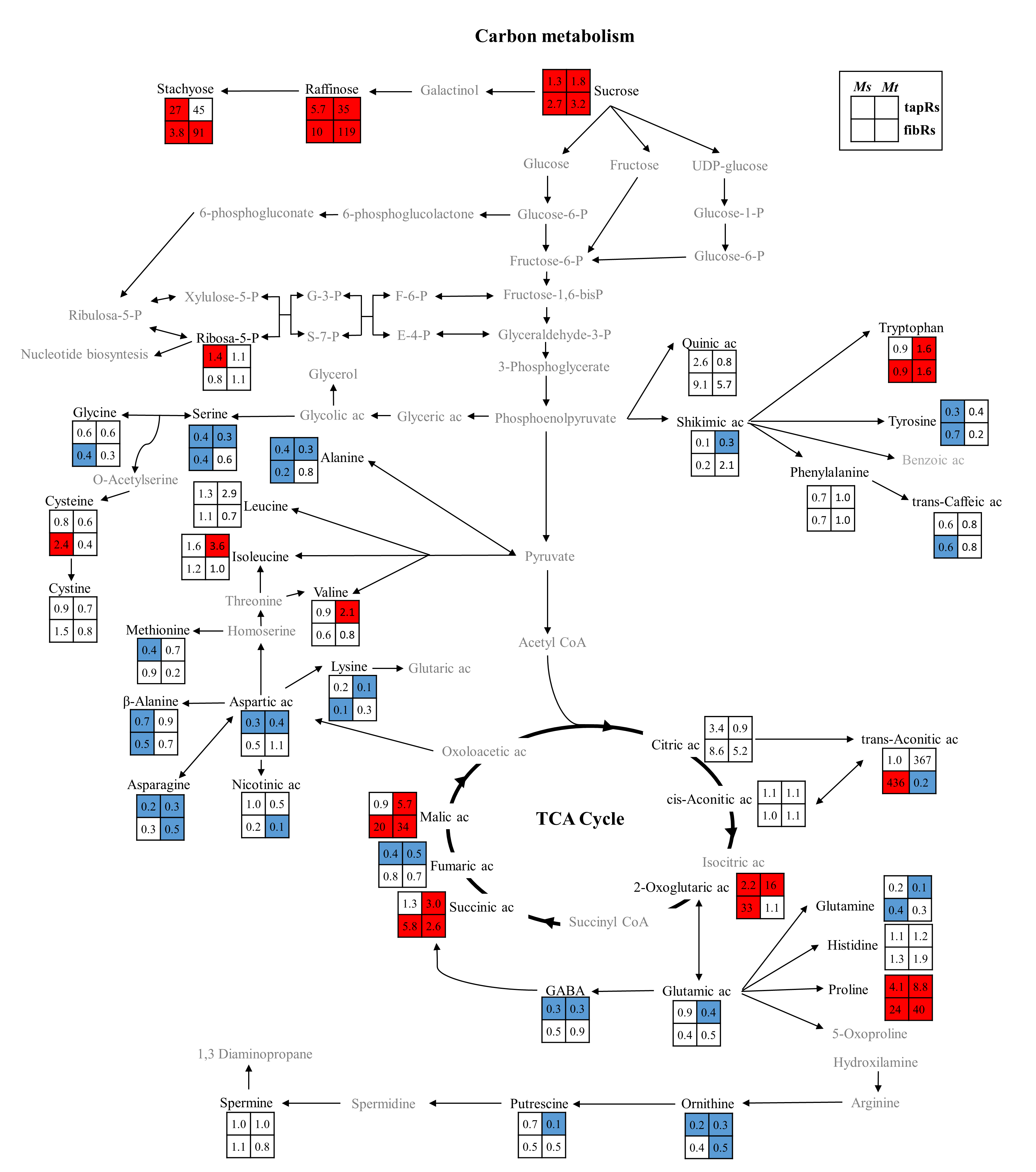

Supplement: Supplementary Figure 4 — Overview of the main metabolic pathways affected under severe water deficit (SD) conditions in Medicago sativa (Ms) and M. truncatula (Mt) in root tissue. Values represented fold-change ratios between SD and control (C) conditions. Significant increase and decrease in the content of a certain metabolite was represented in red and blue, respectively. Metabolites written in gray were not identified in the GC-TOF/MS-based analysis. Ac, acid; GABA, γ-aminobutyric acid; TCA, tricarboxylic acid; UDP, uridine diphosphate; fibrous roots, fibRs; taproots, tapRs. [file Image_4.TIF]

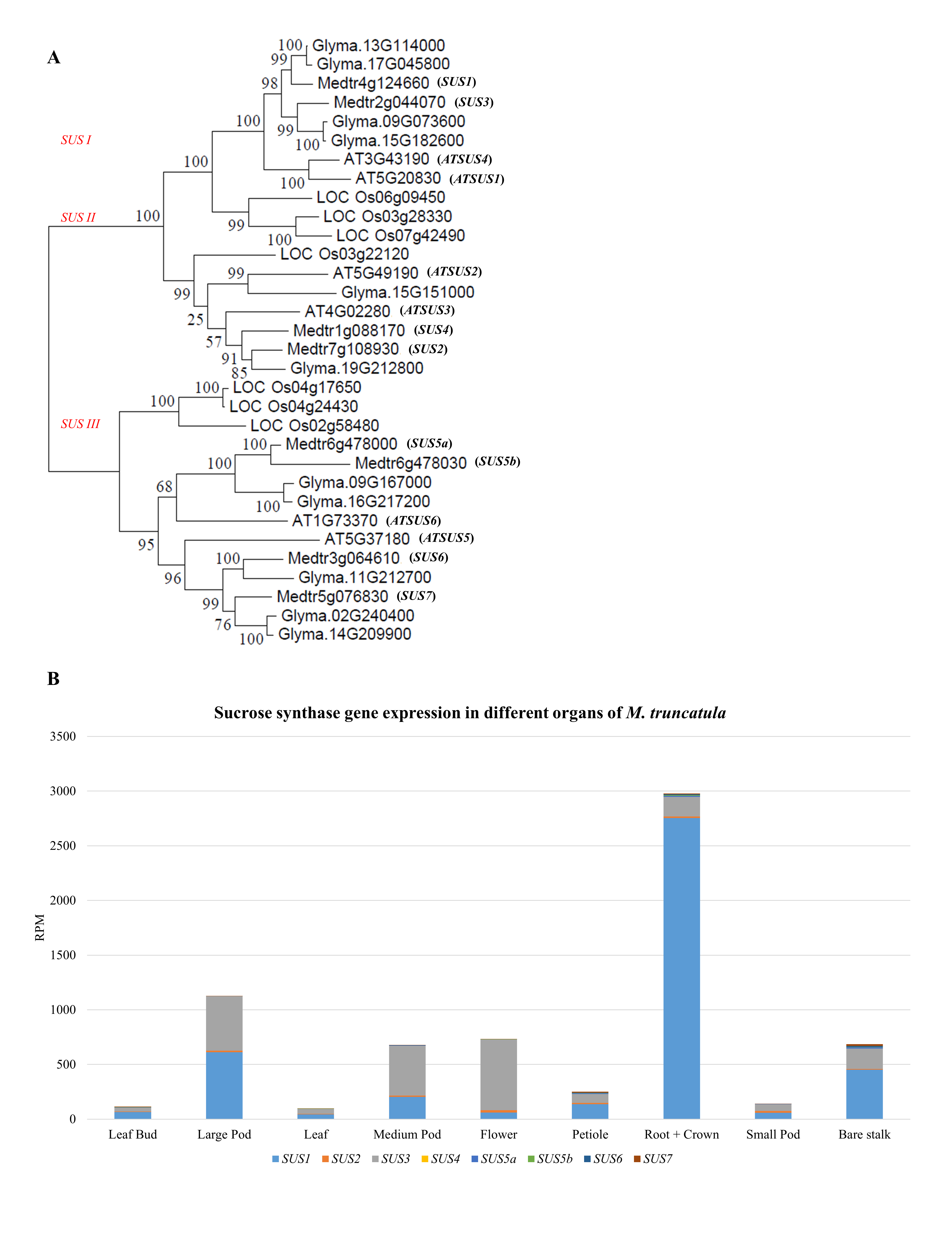

Supplement: Supplementary Figure 5 — (A) Phylogenetic analysis of the sucrose synthase (SUS) family in Medicago truncatula (Mt), Oryza sativa, Glycine max, and Arabidopsis thaliana. A maximum likelihood tree was constructed with MEGA 10.1 (https://www.megasoftware.net/) using the predicted amino acid sequences based on the Whelan And Goldman (WAG) model (Whelan and Goldman, 2001). Values on the branches represented bootstrap values (1,000 repetitions). SUS gene names for Mt and A. thaliana were specified in brackets. (B) Expression levels of the SUS family in Mt based on the RNA-sequencing data extracted from the MtSSPdb database (https://mtsspdb.noble.org/database/). RPM, reads per million. [file Image_5.TIF]
